# Point Mutations in the *FLT3*-ITD Region Are Rare but Recurrent Alterations in Adult AML and Associated With Concomitant *KMT2A*-PTD

**DOI:** 10.3389/fonc.2022.862991

**Published:** 2022-03-21

**Authors:** Sebastian Stasik, Michael Kramer, Sven Zukunft, Christoph Röllig, Claudia D. Baldus, Uwe Platzbecker, Hubert Serve, Carsten Müller-Tidow, Kerstin Schäfer-Eckart, Martin Kaufmann, Stefan Krause, Tim Sauer, Mathias Hänel, Andreas Neubauer, Gerhard Ehninger, Martin Bornhäuser, Johannes Schetelig, Jan M. Middeke, Christian Thiede

**Affiliations:** ^1^ Medizinische Klinik und Poliklinik I, Universitätsklinikum Carl Gustav Carus, Dresden, Germany; ^2^ German Cancer Consortium (DKTK), Partner Site Dresden, and German Cancer Research Center (DKFZ), Heidelberg, Germany; ^3^ Hämatologie und Onkologie, Charité-Universitätsmedizin Berlin, Berlin, Germany; ^4^ Medizinische Klinik und Poliklinik I, Hämatologie und Zelltherapie, Universitätsklinikum Leipzig, Leipzig, Germany; ^5^ Medizinische Klinik II, Universitätsklinikum Frankfurt, Frankfurt am Main, Germany; ^6^ German Cancer Consortium (DKTK), Partner Site Frankfurt, and German Cancer Research Center (DKFZ), Heidelberg, Germany; ^7^ Medizinische Klinik V, Universitätsklinikum Heidelberg, Heidelberg, Germany; ^8^ Klinik für Innere Medizin V, Klinikum Nürnberg Nord, Nürnberg, Germany; ^9^ Abteilung für Hämatologie, Onkologie und Palliativmedizin, Robert-Bosch-Krankenhaus, Stuttgart, Germany; ^10^ Medizinische Klinik V, Universitätsklinikum Erlangen, Erlangen, Germany; ^11^ Medizinische Klinik III, Klinikum Chemnitz, Chemnitz, Germany; ^12^ Klinik für Hämatologie, Onkologie, Immunologie, Philipps Universität Marburg, Marburg, Germany; ^13^ National Center for Tumor Diseases, Dresden, Germany; ^14^ Deutsche Knochenmarkspenderdatei (DKMS) Clinical Trials Unit, Dresden, Germany; ^15^ AgenDix GmbH, Dresden, Germany

**Keywords:** *FLT3*-ITD, point mutations, acute myeloid leukemia (AML), clinical outcome, *KMT2A*-PTD

## Abstract

*FLT3*-ITD mutations are common druggable alterations in patients with acute myeloid leukemia (AML) and associated with poor prognosis. Beside typical ITD mutations, point mutations and deletions in the juxtamembrane domain (JMD) have been observed. However, due to the low frequency of these alterations, there is only limited information on molecular and clinical associations. To evaluate the prognostic impact of non-ITD mutations in the *FLT3* JMD region, we analyzed a large cohort of 1,539 adult AML patients treated in different protocols of the Study Alliance Leukemia, using next-generation sequencing. Non-ITD point mutations and deletions within the *FLT3* JMD were identified with a prevalence of ~1.23% (n = 19). Both *FLT3*-ITD and non-ITD mutations were associated with a higher rate of *NPM1* (42%–61%; *p* < 0.001) and *DNMT3A* mutations (37%–43%; *p* < 0.001), as well as an increased percentage of peripheral blood (54%–65%) and bone marrow blast cells (74%; *p* < 0.001), compared to *FLT3*-wild-type patients. Most significantly, AML patients with *FLT3* non-ITD mutations had a higher rate of concomitant *KMT2A*-PTD mutations (37.5%; *p* < 0.001) as compared to *FLT3*-ITD (7%) or *FLT3*-wild-type cases (4.5%). In a multivariable analysis, *FLT3* non-ITD mutations were not an independent prognostic factor. However, patients with dual *FLT3* non-ITD and *KMT2A*-PTD mutations showed a trend for inferior outcome, which points at a functional interaction in this subset of AML.

## Introduction

The FMS-like tyrosine kinase 3 (FLT3) is a transmembrane receptor tyrosine kinase, which is expressed by hematopoietic stem cells and stimulates the development of myeloid and lymphoid progenitor cells (1). Mutations of the *FLT3* gene are identified in ~30% of patients with acute myeloid leukemia (AML). About 20%–25% of AML patients show internal tandem duplications (*FLT3*-ITD), affecting the juxtamembrane domain (JMD) and/or the tyrosine kinase domain-1 (TKD1) of *FLT3* (2). In addition, mutations in exon 20, coding for the TKD2 region (most frequently affecting codons D835 and I836), can be detected in 7%–10% of AML patients (*FLT3*-TKD) (1, 2).

Both *FLT3*-ITD and *FLT3*-TKD mutations constitutively activate the FLT3 kinase, inducing proliferation of leukemic populations, although differences in the signaling induced have been reported between these two mutations (1). While the clinical significance of *FLT3*-TKD mutations is uncertain, the presence of *FLT3*-ITD mutations in AML patients confers a poor prognosis with an increased risk of relapse and shorter overall survival (2, 3). Consequently, the *FLT3*-ITD mutational status is included in the current risk classification of the ELN-2017 recommendations and is recognized as target for specific TK inhibitors (2, 4, 5). However, several factors modify the prognostic impact of *FLT3*-ITD mutations, such as the allelic ratio and the presence of a concomitant *NPM1* mutation (4).

In addition to typical ITD mutations, previous reports indicated the presence of rare non-ITD mutations (small deletions and point mutations) in the *FLT3* JMD of AML patients (6–16). However, due to the low frequency of these alterations, there are so far only casuistic reports available, with limited information on molecular and clinical associations. To investigate the prevalence and prognostic impact of non-ITD mutations in the *FLT3* JMD of adult patients with AML, we analyzed a large cohort of 1,539 patients with newly diagnosed AML to correlate these mutations with clinical characteristics, co-mutations, and outcome.

## Methods

### Patients

All patients (n = 1,539) investigated had newly diagnosed AML, were registered in clinical protocols of the Study Alliance Leukemia (SAL) (AML96, AML2003 or AML60+, SORAML), and had available biomaterial at diagnosis. Detailed descriptions of the treatment protocols have been published previously (17–20); all protocols included intensive induction chemotherapy and consolidation treatment according to cytogenetic risk groups. The study was in agreement with the Helsinki declaration and approved by the ethical board of the Technical University Dresden (EK98032010).

### Molecular Analysis

Molecular studies were performed on genomic DNA isolated from bone marrow (BM) aspirates or peripheral blood (PB) taken at diagnosis. DNA was extracted using the DNeasy Blood and Tissue Kit (Qiagen, Hilden, Germany) and quantified with the NanoDrop spectrophotometer. In addition to conventional fragment analysis, profiling of *FLT3* JMD/TKD1 mutational status and associated co-mutations was performed by targeted resequencing using the TruSight Myeloid assay (Illumina, San Diego, CA, USA) (**Supplementary Methods**). Briefly, for *FLT3* the panel covers the entire ITD region (AA 572–630). Libraries were sequenced paired-end (150 bp) on a NextSeq instrument (Illumina) and analyzed using the Sequence Pilot Software (JSI medical systems) with a 5% variant allele frequency (VAF) mutation calling cutoff. *KMT2A*-PTD (partial tandem duplication) mutations (formerly *MLL*-PTD) were analyzed on cDNA (reverse transcribed from 1 µg of total RNA; SuperScript VILO cDNA Synthesis Kit; Invitrogen, Carlsbad, CA, USA), using the Mentype AMLplex QS Kit (Biotype, Dresden, Germany) on a 3130xl Genetic Analyzer (Applied Biosystems, Foster, CA, USA).

### Statistical Analysis

Categorical variables between groups were compared using the chi-squared test or a 2-sided Fisher’s exact test. For continuous variables the non-parametric Mann–Whitney U test was applied. *p* values <0.05 were considered significant. To evaluate relapse-free survival (RFS) and overall survival (OS), the Kaplan–Meier method and the log-rank test were used. For multivariable analysis of prognostic factors, Cox-proportional hazard regression models were used for survival endpoints, and logistic regression models were used for CR. All statistical analyses were performed using the R environment for statistical computing version 4.0.3.

## Results

### Characterization of FLT3 Non-ITD Mutations in the JMD/TKD1 Region

**Figure 1:**
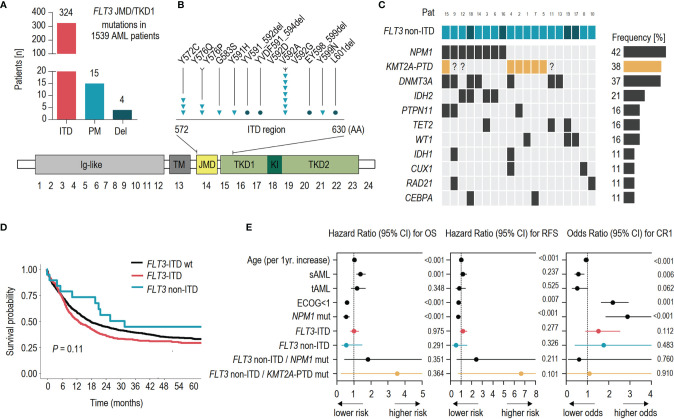
Clinical and molecular associations of *FLT3* non-ITD mutations in AML. **(A)** Frequencies of molecular alterations detected in the *FLT3* JMD/TKD1 region of 1,539 AML patients: ITD, (internal tandem duplication); PM, (point mutation); and Del, (deletion). **(B)** Schematic illustration showing the position of acquired *FLT3* non-ITD mutations (triangles = point mutations, circles = deletions) and the domain structure of *FLT3*: lg-like, (ligand binding domain); TM, (transmembrane domain); JMD, (juxtamembrane domain); TKD1, (tyrosine kinase domain-1); KI, (kinase insert); TKD2, (tyrosine kinase domain-2). **(C)** Associated co-mutations in *FLT3* non-ITD patients. *KMT2A*-PTD data are available for 16/19 patients with *FLT3* non-ITD mutation. Genes with <10% mutation rate are not shown: *EZH2*, *ASXL1*, *IKFZ1*, *NRAS*, *RUNX1*, *SRSF2*, *TP53*. **(D)** Kaplan–Meier analysis showing the probability of overall survival (OS) for AML patients without molecular alterations in the *FLT3*-ITD region (wt; n = 1,196; black), ITD mutation (n = 324; red), and non-ITD mutation (n = 19; blue). **(E)** Results of the multivariable analysis of prognostic factors for overall survival, (OS); relapse-free survival, (RFS) and complete remission, (CR1).

*FLT3*-ITD mutations were detected in 324 of 1,539 (21.1%) AML patients. Non-ITD mutations within the *FLT3* JMD were found in 19 cases (1.23%) (**Figure 1A**). Patients with mutations in the TKD2 region (*FLT3* Exon 20; D835 and I836; n = 104) were excluded from this analysis. Patients without JMD/TKD1 mutation were classified as *FLT3*-ITD wild-type (wt) (n = 1,196). Most non-ITD mutations were single-nucleotide missense variants (SNV; n = 15; **Figure 1B**). Deletions were found in 4 patients, exclusively comprising small in-frame deletions (1–4 amino acid residues). *FLT3* non-ITD mutations were detected with a median VAF of 28% (range 7%–50%) at subclonal levels in the majority of patients (73.7%; **Table S1**). Recurrent point mutations affected residues V592 (n = 7), Y572 (n = 3), and L576 (n = 2) (**Figure 1B**). Compared to *FLT3*-wt patients, both ITD and non-ITD mutations had increased rates of concomitant mutations in *NPM1* (60.7% and 42.1% vs. 23.4%; *p* < 0.001) and *DNMT3A* (43% and 37% vs. 24%; *p* < 0.001; **Table 1** and **Figure 1C**). Vice versa, *FLT3*-wt patients were significantly more often affected by mutations in *NRAS* and *KIT*. No major difference between *FLT3* mutant and -wt patients was observed for other frequently mutated genes in AML, such as *IDH1* or *TET2* (**Table S2**). Notably, non-ITD mutations (exclusively point mutations) were associated with a higher frequency of concomitant *KMT2A*-PTD mutations (37.5% vs. 7% and 4.5%; *p* < 0.001), compared to *FLT3*-ITD or *FLT3*-wt patients (**Table 1** and **Figure 1C**). In addition to *KMT2A*-PTD, other 11q abnormalities were also significantly more frequent in patients with non-ITD variants (**Table 1**).

**Table 1:** Clinical and molecular characteristics of patients.

Parameter	*FLT3*-ITD	*FLT3* non-ITD	*p*-value*^c^*	*FLT3* wt	*p*-value*^d^*
**No. of patients (n)**	324	19		1196	
**Age, years, median (IQR)**	55 (44–64)	51 (45–60)	0.289	56 (45–66)	0.405
**Sex, n/nval (%)**			0.486		**0.049**
Female	169/324 (52.2)	12/19 (63.2)		548/1196 (45.8)	
Male	155/324 (47.8)	7/19 (36.8)		648/1196 (54.2)	
**Disease status, n/nval (%)**			**0.045**		**0.004**
*De novo*	290/322 (90.1)	17/19 (89.5)		974/1180 (82.5)	
sAML	25/322 (7.8)	0/19 (0)		155/1180 (13.1)	
tAML	7/322 (2.2)	2/19 (10.5)		51/1180 (4.3)	
**ELN-Risk 2017, n/nval (%)**			0.279		**<0.001**
Favorable	89/298 (29.9)	2/15 (13.3)		447/1109 (40.3)	
Intermediate	123/298 (41.3)	9/15 (60)		238/1109 (21.5)	
Adverse	86/298 (28.9)	4/15 (26.7)		424/1109 (38.2)	
**Normal karyotype, n/nval (%)**			**0.003**		**<0.001**
No	77/303 (25.4)	10/16 (62.5)		568/1125 (50.5)	
Yes	226/303 (74.6)	6/16 (37.5)		557/1125 (49.5)	
**Complex karyotype, n/nval (%)**			1.000		**<0.001**
No	297/305 (97.4)	17/17 (100)		908/1083 (83.8)	
Yes	8/305 (2.6)	0/17 (0)		175/1083 (16.2)	
**t(11;v), n/nval (%)**			**<0.001**		**0.001**
No	289/291 (99.3)	10/12 (83.3)		989/1018 (97.2)	
Yes	2/291 (0.7)	2/12 (16.7)		29/1018 (2.8)	
**ECOG score, n/nval (%)**			0.646		**0.021**
0–1	170/266 (63.9)	13/18 (72.2)		717/987 (72.6)	
2–4	96/266 (36.1)	5/18 (27.8)		270/987 (27.4)	
**Laboratory, median (IQR)**					
WBC (Gpt/l)	46.09 (17.1–100.65)	39.6 (13.44–63.5)	0.527	13.3 (3.59–40.68)	**<0.001**
Hemoglobin (mmol/l)	5.77 (5.01–6.77)	5.34 (5.25–5.9)	0.653	5.9 (5.03–7.09)	0.165
PLT (Gpt/l)	58 (30.5–102.5)	42 (29.5–89)	0.474	48 (26–92)	**0.028**
Peripheral blasts (%)	63 (25–84)	54 (20–86.5)	0.960	33 (9–66)	<0.001
LDH (U/l)	602.4 (389–964)	591 (432–859)	0.757	411 (255–692.25)	**<0.001**
BM blasts (%)	74 (57.36–85)	74 (64.25–83.5)	0.539	59 (41.5–74.5)	**<0.001**
**Frequent co-mutations, n/nval (%)**					
*KMT2A*-PTD	9/128 (7)	6/16 (37.5)	**0.001**	19/424 (4.5)	**<0.001**
*NPM1*	196/323 (60.7)	8/19 (42.1)	0.173	279/1191 (23.4)	**<0.001**
*DNMT3A*	139/324 (43)	7/19 (37)	0.779	282/1196 (24)	**<0.001**
*IDH2*	30/324 (9)	4/19 (21)	0.202	186/1196 (16)	**0.011**
*WT1*	40/324 (12)	3/19 (16)	0.933	68/1196 (6)	**<0.001**
*KIT*	5/324 (2)	0/19 (0)	1.000	70/1196 (6)	**0.004**
*NRAS*	30/324 (9)	1/19 (5)	0.858	211/1196 (18)	**<0.001**
**Clinical outcome** *^a^*					
Follow-up, median (IQR) in months	100 (70–116)	108 (80–127)		86 (40–115)	
CR rate, n (%)	252 (77.8)	16 (85)	0.513	818 (68.4)	**0.001**
RFS, median (95% CI) in months*^b^*	9.69 (7.98–14.2)	18.5 (13.8–NA)	0.181	22.8 (18.3–27.4)	**0.001**
OS, median (95% CI) in months	13.1 (10.5–17.6)	31.7 (19.8–NA)	0.109	17.9 (15.9–21.4)	0.107

Significant differences are presented in bold.

^a^Results of the univariate logistic regression model for CR1 and Cox regression model for RFS/OS; NA means infinity.

^b^The median RFS is calculated with the positively selected subgroup of patients with a CR.

^c^p-values for the differences between FLT3-ITD and FLT3 non-ITD patients.

^d^p-values for the comparison of all three groups (FLT3-ITD, FLT3 non-ITD and FLT3 wt).

tAML, therapy-related acute myeloid leukemia; sAML, secondary acute myeloid leukemia; WBC, white blood cells; BM, bone marrow; PB, peripheral blood; LDH, lactate dehydrogenase; ELN, European LeukemiaNet; OS, overall survival; RFS, relapse-free survival; CR, complete remission; ED30, early death within 30 days; EFS, event-free survival; n number; nval, number of valid non-missing observations; IQR, interquartile range; 95% CI, 95% confidence interval.

### Associations of FLT3 Non-ITD Mutations With Clinical Features and Outcome

For patients with *FLT3* non-ITD mutations, the median follow-up was 108 months (IQR 80–127 months). The first complete remission (CR1) after initial treatment was achieved in 16/19 (84.2%) patients. The relapse rate after CR1 was 62.5% (10/16). Of these patients, 20% (2/10) achieved a second CR. The comparison between patients with *FLT3* non-ITD and ITD variants revealed significant similarities (as compared to *FLT3*-wt patients) but also several differences of clinical parameters (**Table 1**). Patients with non-ITD mutations (median age 51; range 45–60 years) were diagnosed significantly more often with tAML (10.5% vs. 2.2%; *p* = 0.045) and had a higher rate of karyotype aberrations compared to *FLT3*-ITD patients (62.5% vs. 25.4%; *p* = 0.003). No differences were detected for the relative proportion of ELN-2017 adverse risk, the ECOG performance status, or the presence of a complex aberrant karyotype between both groups. Likewise, general similarities were observed for most laboratory parameters, such as WBC counts (median 39.6–46.09 Gpt l^-1^), PB (median 54–63 Gpt l^-1^), and BM blasts (median 74%). In contrast, *FLT3*-wt status was associated with a higher rate of ELN-2017 adverse risk (38.2%; *p* < 0.001) and complex aberrant karyotype (16.2%; *p* < 0.001), as well as lower WBC counts and lower rates of PB and BM blasts compared to *FLT3* mutant patients. With respect to clinical outcome, *FLT3* non-ITD mutations were not associated with CR rate (85%), relapse-free survival (median 18.5 months), or overall survival (median 31.7 months) in univariate analyses (**Figure 1D** and **Table 1**). Likewise, the multivariable analysis revealed that non-ITD mutations (as well as *FLT3*-ITD mutations) were not an independent prognostic factor for outcome (**Figure 1E** and **Table S3**). Interestingly, there was an insignificant trend toward inferior RFS (HR 6.64; CI 95% 0.691–63.9; *p* = 0.101; **Figure 1E**) for patients with the mutual presence of *FLT3* non-ITD and *KMT2A*-PTD mutations (**Figure S1** and **Table S3**). Regarding the effect of transplantation on outcome, only two (out of 19) patients with *FLT3* non-ITD mutations underwent allogeneic hematopoietic stem cell transplantation (alloHSCT) without subsequent event (**Figure S2**). Thus, compared to *FLT3*-ITD and -wt patients, no significant differences were observed.

## Discussion

In the present study, we analyzed 1,539 adult AML patients for the prevalence and prognostic impact of non-ITD mutations in the JMD/TKD1 region of *FLT3*. We confirm that JMD point mutations and deletions are rare, but recurrent alterations in patients with AML at a frequency in the range of previous estimates (8, 9). Compared to AML, higher rates (~4-fold) of JMD non-ITD mutations were previously detected in patients with acute lymphoblastic leukemia (ALL) (8). Among non-ITD mutations in our cohort, the point mutations Y572C, V592G (9), L576Q (10), G583S (8), Y591H (11), and V592A (12, 13) as well as the deletion EY598_599del (14) were previously recognized as gain-of-function mutations that result in constitutive kinase activation and stimulate AML growth through aberrant STAT5 signaling (9, 12, 14). In addition, in one patient we identified a novel and likely damaging (PolyPhen score = 1) point mutation at Y599N (30% VAF), not previously reported in the literature. However, functional data indicate that any alteration of the JMD sequence interferes with the kinase auto-inhibition, similar to the effect of *FLT3*-ITD mutations (9, 11, 15). Accordingly, *in vitro* studies illustrated the response of AML cells with non-ITD JMD mutations to pharmacologic FLT3 inhibition (9, 10, 12, 14–16), which supports the idea to use any of the approved TKIs in patients with this type of mutation (5). In line with this, we demonstrate high similarities between patients with *FLT3*-ITD and non-ITD mutations, with respect to major laboratory parameters (i.e., an increased percentage of blast cells in PB and BM) and the rate of co-mutated driver variants, which is consistent with the mutational landscape and clinical phenotype typically observed for patients with *FLT3*-ITD mutations (1–3, 21). For example, a high rate of concomitant *NPM1* and *DNMT3A* mutations but mutual exclusivity with downstream effectors such as *NRAS* in *FLT3* mutant patients has been described in detail (21). Similar to our data, partial tandem duplications (PTD) of the histone methyltransferase *KMT2A* (MLL) occur in 3%–5% of AML cases and are typically enriched in patients with *FLT3*-ITD mutations (as compared to *FLT3*-wt) (21, 22). In addition, we show a not previously reported high association of *KMT2A*-PTD mutations with *FLT3* JMD point mutations, which adds on initial observations on the genomic landscape of *KMT2A*-PTD-mutated AML (22). More importantly, although the presence of *FLT3* non-ITD mutations alone was not an independent prognostic factor, patients with dual non-ITD and *KMT2A*-PTD mutations showed a trend for inferior outcome. While *KMT2A*-PTD mutations are typically associated with poor prognosis, isolated *KMT2A*-PTD mutations are not sufficient to establish leukemic transformation of hematopoietic cells, highlighting the importance of cooperating genetic lesions, such as *FLT3* mutations in KMT2A-rearranged leukemias (22–24). Likewise, functional data indicate the transcriptional regulation of *FLT3* by KMT2A *via* aberrant *MEIS1* gene expression (25). More recently, the combined inhibition of menin-MLL (*MLL1*, *KMT2A*) and FLT3 demonstrated a synergistic therapeutic opportunity in these leukemia subtypes (26). However, mutations of genes associated with RAS signaling (i.e., *FLT3*) may be frequently lost or emerge during AML progression, with relevance for post-remission strategies (27). In this regard, future investigations are needed to evaluate the impact of *FLT3* non-ITD mutations for relapse development. In conclusion, we show that *FLT3* non-ITD mutations are rare but recurrent alterations in AML and associated with similar clinical features like FLT3-ITD variants. Interestingly, our data point at a functional interaction of *FLT3* non-ITD mutations with *KMT2A*-PTD and 11q-abnormalities as cooperating genetic events, which might be indicative of a distinct pathway of genesis in this subset of AML.

## Data Availability Statement

The datasets presented in this article are not readily available because patients did not consent to have data uploaded to a public database. Requests to access the datasets should be directed to the corresponding author on reasonable request: christian.thiede@uniklinikum-dresden.de.

## Ethics Statement

The studies involving human participants were reviewed and approved by Technical University Dresden (EK98032010). The patients/participants provided their written informed consent to participate in this study.

## Author Contributions

Conception of the work: CT, SS. Sample/Data Collection: All authors. Aquisition/Analysis of Data: CT, SS. Bioinformatic Analysis: SS. Statistical Analysis: MK, SZ. Interpretation of Data: CT, SS. Drafted the manuscript: SS. Administrative support: GE, MB. All authors have read and approved the manuscript being submitted.

## Conflict of Interest

CT is CEO and co-owner of AgenDix GmbH, a company performing molecular diagnostics.

The authors declare that the research was conducted in the absence of any commercial or financial relationships that could be construed as a potential conflict of interest.

## Publisher’s Note

All claims expressed in this article are solely those of the authors and do not necessarily represent those of their affiliated organizations, or those of the publisher, the editors and the reviewers. Any product that may be evaluated in this article, or claim that may be made by its manufacturer, is not guaranteed or endorsed by the publisher.
