# Supplementary material for: Point Mutations in the FLT3-ITD Region Are Rare but Recurrent Alterations in Adult AML and Associated With Concomitant KMT2A-PTD
Source: Front Oncol. 2022 Mar 21;12:862991. doi: 10.3389/fonc.2022.862991 (PMC8977490; doi:10.3389/fonc.2022.862991)
Supplement: Supplementary file 1 [file DataSheet_1.docx]

**Supplementary Apendix**

**Point mutations in the *FLT3*-ITD region are rare but recurrent alterations in adult AML and associated with concomitant *KMT2A*-PTD**

(Stasik et al.)

**Supplementary Methods**

*DNA preparation and targeted sequencing using NGS*

Profiling of *FLT3* mutational status and associated co-mutations was performed by targeted resequencing using the TruSight Myeloid assay (Illumina) covering 54 genes frequently mutated in AML: *BCOR*, *BCORL1*, *CDKN2A*, *CEBPA*, *CUX1*, *DNMT3A*, *ETV6*, *EZH2*, *IKZF1*, *KDM6A*, *PHF6*, *RAD21*, *RUNX1*, *STAG2*, *ZRSR2*, *ABL1*, *ASXL1*, *ATRX*, *BRAF*, *CALR*, *CBL*, *CBLB*, *CBLC*, *CDKN2A*, *CSF3R*, *FBXW7*, *FLT3*, *GATA1*, *GATA2*, *GNAS*, *HRAS*, *IDH1*, *IDH2*, *JAK2*, *JAK3*, *KIT*, *KRAS*, *KMT2A*, *MPL*, *MYD88*, *NOTCH1*, *NPM1*, *NRAS*, *PDGFRA*, *PTEN*, *PTPN11*, *SETBP1*, *SF3B1*, *SMC1A*, *SMC3*, *SRSF2*, *TET2*, *TP53*, *U2AF1* and *WT1*. For each reaction, 50 ng of genomic DNA was used. Library preparation was done as recommended by the manufacturer (TruSight Myeloid Sequencing Panel Reference Guide 15054779 v02, Illumina). Samples were sequenced paired-end (150bp PE) on a NextSeq NGS-instrument (Illumina). Sequence data alignment of demultiplexed FastQ files, variant calling and filtering was done using the Sequence Pilot software package (JSI medical systems GmbH, Ettenheim, Germany) with default settings and a 5% variant allele frequency (VAF) mutation calling cut-off. Human genome build HG19 was used as reference genome for mapping algorithms. In addition to NGS, conventional fragment analysis was used for profiling of *FLT3*-ITD mutations in all samples, as described previously [1]. Clonality was determined by the comparison of detected *FLT3* allelic ratios with VAFs of co-mutated driver variants, as described previously [2].

**Supplementary Data**

| **Table S1: Characteristics of patients with *FLT3* non-ITD mutations** | | | | | | | | | |  |
| --- | --- | --- | --- | --- | --- | --- | --- | --- | --- | --- |
| **Pat** | **Mutation** | | **VAF [%]** | **Clonality** | ***KMT2A*-PTD** | | **Karyotype** | | | **ELNRisk** |
| 1 | p.Val592Ala | | 24 | Subclonal | mut | | 46,XY | | | adv |
| 2 | p.Leu576Gln | | 12 | Dominant | mut | | 46,XY,?t(X;11)(q2?4;q23),del(9)(q13q22) | | | adv |
| 3 | p.Tyr591His | | 50 | Dominant | wt | | 46,XX | | | int |
| 4 | p.Val592Asp | | 41 | Subclonal | mut | | 47,XX,+11 | | | int |
| 5 | p.Val592Ala | | 31 | Dominant | mut | | 46,XX,t(2;15)(p1?3;q2?2) | | | int |
| 6 | p.Tyr599Asn | | 30 | Subclonal | wt | | 46,XX | | | int |
| 7 | p.Tyr572Cys | | 10 | Subclonal | mut | | 46,XX,+mar | | | int |
| 8 | p.Val592Ala | | 7 | Subclonal | wt | | 46,XX,inv(16)(p13q22),t(16;16)(p13;q22) | | | fav |
| 9 | p.Tyr572Cys | | 19 | Subclonal | n.a. | | 46,XX | | | int |
| 10 | p.Gly583Ser | | 30 | Subclonal | wt | | n.a. | | | n.a. |
| 11 | p.Tyr572Cys | | 45 | Subclonal | n.a. | | 46,XY,t(2;14)(q?2;q32) | | | adv |
| 12 | p.Val592Ala | | 28 | Subclonal | n.a. | | n.a. | | | n.a. |
| 13 | p.Val592Gly | | 21 | Subclonal | wt | | 47,XY,+8,t(9;11)(p22;q23) | | | int |
| 14 | p.Leu576Pro | | 30 | Subclonal | wt | | n.a. | | | n.a. |
| 15 | p.Val592Asp | | 13 | Subclonal | mut | | 46,XX | | | fav |
| 16 | p.Glu598_Tyr599del | | 38 | Dominant | wt | | 47,XX,?+i(8)(q10) | | | adv |
| 17 | p.Tyr591_Phe594del | | 6 | Dominant | wt | | 47,XX,+4 | | | int |
| 18 | p.Asp600_Leu601del | | 36 | Subclonal | wt | | 47,XY,+4 | | | int |
| 19 | p.Phe590_Val592del | | 8 | Subclonal | wt | | 46,XY,inv(16)(p13q22) | | | fav |
| Abbreviations: ELN (European LeukemiaNET), adv (adverse), int (intermediate), fav (favorable), OS (Overall survival), RFS (Relapse-free survival), CR (Complete Remission), n.a. (not available) | | | | | | | | | | |
|  |  |  |  |  |  |  |  |  |  |  |
| **Table S2: Frequencies of co-mutated variants in AML patients** | | | | | | | | |  |  |
| **Gene** **mut, n/nval (%)** | | ***FLT3*-ITD** | | | | ***FLT3* non-ITD** | | ***FLT3*-ITD wt** |  |  |
| *ASXL1* | | 11/324 (3) | | | | 1/19 (5) | | 111/1196 (9) |  |  |
| *BCOR* | | 7/324 (2) | | | | 0/19 (0) | | 66/1196 (6) |  |  |
| *BCORL1* | | 12/324 (4) | | | | 0/19 (0) | | 44/1196 (4) |  |  |
| *CEBPA* | | 40/313 (13) | | | | 2/19 (11) | | 211/1156 (18) |  |  |
| *EZH2* | | 7/324 (2) | | | | 1/19 (5) | | 51/1196 (4) |  |  |
| *IDH1* | | 34/324 (10) | | | | 2/19 (11) | | 105/1196 (9) |  |  |
| *PTPN11* | | 20/324 (6) | | | | 3/19 (16) | | 88/1196 (7) |  |  |
| *RAD21* | | 14/324 (4) | | | | 2/19 (11) | | 34/1196 (3) |  |  |
| *RUNX1* | | 30/324 (9) | | | | 1/19 (5) | | 106/1196 (9) |  |  |
| *SRSF2* | | 7/324 (2) | | | | 1/19 (5) | | 94/1196 (8) |  |  |
| *TET2* | | 59/324 (18) | | | | 3/19 (16) | | 231/1196 (19) |  |  |
| *TP53* | | 3/324 (1) | | | | 1/19 (5) | | 104/1196 (9) |  |  |

| **Table S3: Results of the multivariable analysis** | |  |  |  |  |  |
| --- | --- | --- | --- | --- | --- | --- |
| **Parameter** | **OS (HR,**  **95%-CI)** | ***P*-value** | **RFS (HR,**  **95%-CI)** | ***P*-value** | **CR1 (OR,**  **95%-CI)** | ***P*-value** |
| age (per 1 yr. increase) | 1.033  (1.027 to 1.038) | <0.001 | 1.023  (1.017 to 1.030) | <0.001 | 0.945  (0.935 to 0.955) | <0.001 |
| sAML | 1.383  (1.135 to 1.686) | 0.001 | 1.184  (0.894 to 1.568) | 0.237 | 0.581  (0.395 to 0.854) | 0.006 |
| tAML | 1.184  (0.832 to 1.685) | 0.348 | 0.84  (0.491 to 1.436) | 0.525 | 0.530  (0.272 to 1.032) | 0.062 |
| ECOGCAT<1 | 0.595  (0.514 to 0.689) | <0.001 | 0.769  (0.635 to 0.930) | 0.007 | 2.193  (1.637 to 2.937) | <0.001 |
| *NPM1* mut | 0.564  (0.459 to 0.692) | <0.001 | 0.62  (0.493 to 0.779) | <0.001 | 2.839  (1.878 to 4.294) | <0.001 |
| *FLT3*-ITD mut | 1.004  (0.782 to 1.287) | 0.975 | 1.177  (0.878 to 1.579) | 0.277 | 1.520  (0.907 to 2.547) | 0.112 |
| *FLT3* non-ITD | 0.621  (0.257 to 1.502) | 0.291 | 0.609  (0.227 to 1.637) | 0.326 | 1.823  (0.340 to 9.760) | 0.483 |
| *FLT3* non-ITD/ *NPM1* mut | 1.886  (0.498 to 7.145) | 0.351 | 2.450  (0.601 to 9.999) | 0.211 | 0.649  (0.041 to 10.30) | 0.760 |
| *FLT3* non-ITD / *KMT2A*-PTD mut | 3.539  (0.231 to 54.12) | 0.364 | 6.646  (0.691 to 63.92) | 0.101 | 1.101  (0.048 to 20.83) | 0.910 |
| Abbreviations: tAML (Therapy-related acute myeloid leukemia), sAML (Secondary acute myeloid leukemia), HR (Hazard ratio), 95%-CI (95% confidence interval), OR (Odds ratio) | | | | | | |

**Supplementary Figures**


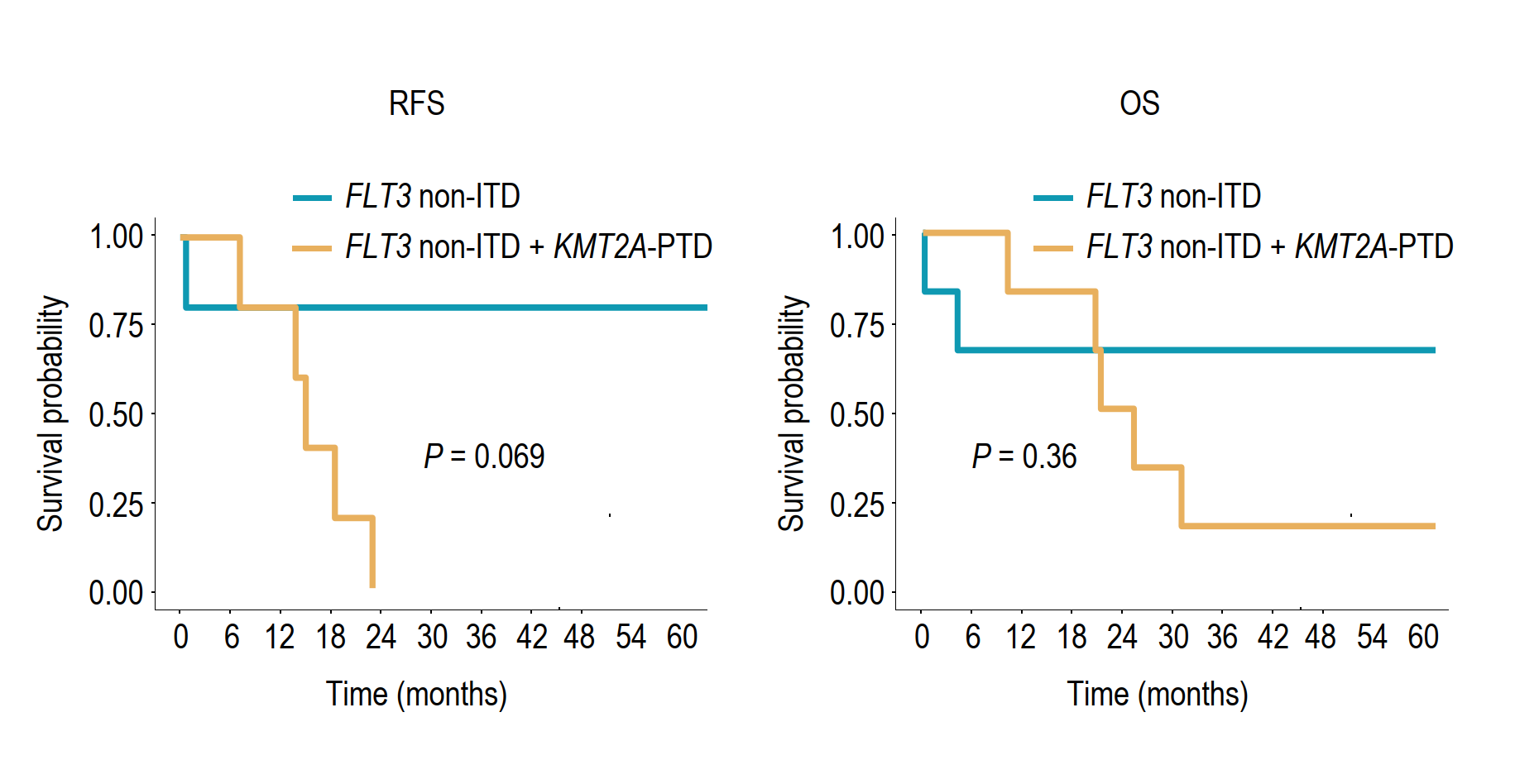


Figure S1

Kaplan-Meier analysis showing the probability of relapse-free survival (RFS) and overall survival (OS) for AML patients with *FLT3* non-ITD mutations, with (yellow) or without (blue) concomitant *KMT2A*-PTD mutation.

**
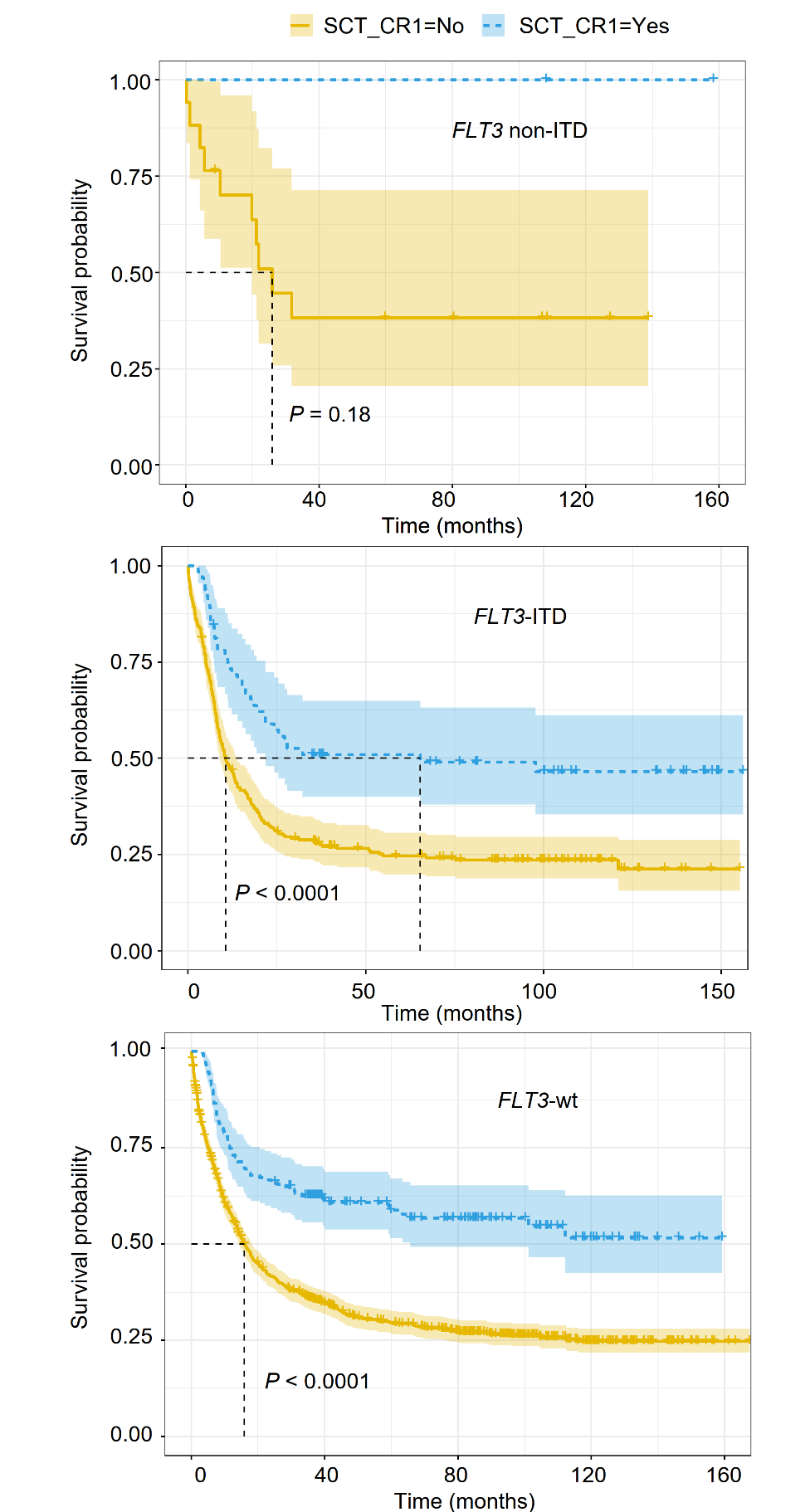
**

Figure S2

Kaplan-Meier analysis showing the probability of overall survival (OS) for AML patients with or without allogeneic haematopoietic stem cell transplantation (alloHSCT) in CR1. Data are shown for the groups (top to bottom) of *FLT3* non-ITD, *FLT3*-ITD, and *FLT3*-wt patients.

**Supplementary References**

[1] Thiede C, Steudel C, Mohr B, Schaich M, Schäkel U, Platzbecker U, Wermke M, Bornhäuser M, Ritter M, Neubauer A, Ehninger G, Illmer T. Analysis of FLT3-activating mutations in 979 patients with acute myelogenous leukemia: association with FAB subtypes and identification of subgroups with poor prognosis. Blood. 2002 Jun 15;99(12):4326-35. doi: 10.1182/blood.v99.12.4326.

[2] Stasik S, Eckardt JN, Kramer M, Röllig C, Krämer A, Scholl S, Hochhaus A, Crysandt M, Brümmendorf TH, Naumann R, Steffen B, Kunzmann V, Einsele H, Schaich M, Burchert A, Neubauer A, Schäfer-Eckart K, Schliemann C, Krause S, Herbst R, Hänel M, Frickhofen N, Noppeney R, Kaiser U, Baldus CD, Kaufmann M, Rácil Z, Platzbecker U, Berdel WE, Mayer J, Serve H, Müller-Tidow C, Ehninger G, Bornhäuser M, Schetelig J, Middeke JM, Thiede C; Study Alliance Leukemia (SAL). Impact of PTPN11 mutations on clinical outcome analyzed in 1529 patients with acute myeloid leukemia. Blood Adv. 2021 Sep 14;5(17):3279-3289. doi: 10.1182/bloodadvances.2021004631.
